# Prevalence and hematological indicators of G6PD deficiency in malaria-infected patients

**DOI:** 10.1186/s40249-016-0130-0

**Published:** 2016-04-25

**Authors:** Manas Kotepui, Kwuntida Uthaisar, Bhukdee PhunPhuech, Nuoil Phiwklam

**Affiliations:** Medical Technology Program, School of Allied Health Sciences and Public Health, Walailak University, Nakhon Si Thammarat, 80161 Thailand; Medical Technology Laboratory, Phop Phra Hospital, Phop Phra District, Tak Province 63160 Thailand

**Keywords:** Malaria, G6PD deficiency, *Plasmodium falciparum*, *Plasmodium vivax*, Thailand

## Abstract

**Background:**

This study aimed to evaluate the prevalence and alteration of hematological parameters in malaria patients with a glucose-6-phosphate dehydrogenase (G6PD) deficiency, in the western region of Thailand, an endemic region for malaria.

**Methods:**

Data about patients with malaria hospitalized between 2013 and 2015 were collected. Clinical and sociodemographic characteristics such as age and gender, diagnosis on admission, and parasitological results were mined from medical records of the laboratory unit of the Phop Phra Hospital in Tak Province, Thailand. Venous blood samples were collected at the time of admission to hospital to determine G6PD deficiency by fluorescence spot test and detect malaria parasites by thick and thin film examination. Other data such as complete blood count and parasite density were also collected and analyzed.

**Results:**

Among the 245 malaria cases, 28 (11.4 %) were diagnosed as *Plasmodium falciparum* infections and 217 cases (88.6 %) were diagnosed as *P. vivax* infections. Seventeen (6.9 %) patients had a G6PD deficiency and 228 (93.1 %) patients did not have a G6PD deficiency. Prevalence of male patients with G6PD deficiency was higher than that of female patients (*P* < 0.05, *OR* = 5.167). Among the patients with a G6PD deficiency, two (11.8 %) were infected with *P. falciparum,* while the remaining were infected with *P. vivax*. Malaria patients with a G6PD deficiency have higher monocyte counts (0.6 × 10^3^/μL) than those without a G6PD deficiency (0.33 × 10^3^/μL) (*P* < 0.05, *OR* = 5.167). Univariate and multivariate analyses also confirmed that malaria patients with a G6PD deficiency have high monocyte counts. The association between G6PD status and monocyte counts was independent of age, gender, nationality, *Plasmodium* species, and parasite density (*P* < 0.005).

**Conclusion:**

This study showed a prevalence of G6PD deficiency in a malaria-endemic area. This study also supported the assertion that patients with G6PD-deficient red blood cells had no protection against the *P. falciparum* infection. In addition, malaria patients with a G6PD deficiency had higher monocyte counts than those without a G6PD deficiency. These findings will help to recognize and diagnose malaria patients with a G6PD deficiency, as well as to identify the risks and protective factors against malaria in endemic areas.

**Electronic supplementary material:**

The online version of this article (doi:10.1186/s40249-016-0130-0) contains supplementary material, which is available to authorized users.

## Multilingual Abstracts

Please see Additional file 1 for translations of the abstract into the five official working languages of the United Nations.

## Background

Glucose-6-phosphate dehydrogenase (G6PD) is an enzyme involved in the production of the NADPH required to protect cells against oxidative stress [1]. A G6PD deficiency is an X-linked hereditary enzyme deficiency affecting approximately 400 million people worldwide, especially in malaria-endemic areas [2]. As a result, G6PD-deficient red blood cells (RBCs) are more susceptible to being destroyed by oxidative stress such as oxidative food (fava beans) and drugs (e.g., primaquine and sulfones). Therefore, this disease is often manifested as acute hemolytic anemia (AHA) triggered by the intake of those stressors [3]. The mutations of the *G6PD* gene result in protein variants with different levels of enzyme activity that are associated with a wide range of biochemical and clinical phenotypes [4]. At present, 186 mutations affecting the gene coding sequence have been reported, with most of these single-base substitutions leading to amino acid replacements [5].

Identifying G6PD deficiency in individuals prior to treatment of malaria is essential to protect patients from hemolytic reactions because malaria and G6PD deficiency share similar geographical distributions. Treatment of malaria patients with antimalarial drugs, such as primaquine or other 8-aminoquinolines, may be associated with potential hemolytic anemia [6]. The most recent study showed that adding primaquine to malaria treatments reduces gametocyte prevalence in patients and may prevent people from transmitting malaria [7]. It is also postulated that G6PD deficiencies have become more frequent because G6PD-deficient variants confer some protection or resistance against malaria caused by the species *P. falciparum* and *P. vivax* [8]. The protective role of a G6PD deficiency against infection with *P. falciparum* malaria has also been supported by various case–control studies [9–11].

The Thailand-Myanmar border area is in the western part of Thailand. It is the most common destination for ethnic minorities from Myanmar who migrate to Thailand for agricultural work. The Thai-Myanmar border is also a malaria-endemic area and is often described as an ‘epicenter’ of drug-resistant malaria. This study aimed to identify the prevalence of G6PD deficiency among patients with malaria and the alteration of hematological parameters in patients with a G6PD deficiency.

## Methods

This study gathered data about patients diagnosed with a malaria infection who have a G6PD deficiency, from the laboratory unit of the Phop Phra Hospital in Tak Province, Thailand, between January 2013 and April 2015. The data were collected by examining the history of malaria patients and the results of G6PD deficiency tests. Other general characteristics about the patients such as age, gender, and nationality were also collected.

The fluorescence spot test (R&D Diagnostics, Aghia Paraskevi, Greece) was conducted to detect the presence or absence of a G6PD deficiency, with the results indicating ‘heterozygous or homozygous G6PD deficiency’ when there wasn’t any signal from the test. Those with fluorescent signals were classified as ‘normal.’ The laboratory data collected comprised complete blood count (CBC) using the BC-5200 Haematology Analyzer (Mindray, Nanshan, Shenzhen, China). This included white blood cell (WBC) count and differential count, RBC count, hemoglobin (Hb) level, platelet counts, mean corpuscular volume (MCV), mean corpuscular hemoglobin (MCH), mean corpuscular hemoglobin concentration (MCHC), and red cell distribution width (RDW). A malaria test was performed by staining a blood film with Giemsa stain. The microscopic abnormality of blood in a smear was detected using a haematology analyzer, and laboratory technicians determined the presence or absence of malaria under a light microscope. The parasite density was also determined and defined as low parasitemia (1–100 parasites/100 oil fields), moderate parasitemia (1–10 parasites/1 oil fields), and high parasitemia (>100 parasites/1 oil field).

Data analysis was performed to show frequency, percentage, and central tendency of patients with malaria. Tests for the homogeneity of data were performed to identify a suitable statistical analysis. Comparisons of proportions were based on the chi-square test or Fisher’s exact test. Tests for the comparison of continuous variables in different groups were based on the independent t-test or Mann–Whitney U test. To test whether there was an association between G6PD status and hematological parameters, a logistic regression was used to adjust for age, gender, nationality, type of malaria, and parasite density. All tests were performed using the SPSS software version 11.5 for Windows (SPSS Inc., Chicago, IL, USA), with *P*-values less than 0.05 considered statistically significant.

The protocol of this study was approved by the Phop Phra Hospital and the Ethical Clearance Committee on Human Rights Related to Researches Involving Human Subjects of Walailak University, Thailand.

## Results

From January 2013 to April 2015, 245 individuals were diagnosed with a malaria infection at the Phop Phra Hospital, Thailand. Of those, 17 (6.9 %) were diagnosed with a G6PD deficiency. The median age was 20 years in patients with a G6PD deficiency and 19 years in patients without a G6PD deficiency. One hundred and fifty cases (61.2 %) were male and 95 (38.8 %) were female. One hundred and thirty cases (53.1 %) were of Thai nationality and 115 (46.9 %) were Burmese. No significant differences were observed between the patients’ age, nationality, or G6PD status in patients with a G6PD deficiency and patients without a G6PD deficiency (*P* > 0.05) (see Table 1). Twenty-eight cases (11.4 %) were infected with *P. falciparum*, while the remaining 217 cases (88.6 %) were infected with *P. vivax.* Of the patients with a G6PD deficiency, two cases (11.8 %) were infected with *P. falciparum* and 15 cases (88.2 %) were infected with *P. vivax*.

**Table 1:** General characteristics of the study participants

Demographic	G6PD status		*P*-value	*OR* (95 % *CI*)
	G6PD def.	Normal		
Age (median, range)	20 (12–52)	19 (0–71)	0.364*	NA
Male, n (%)	15 (88.2 %)	135 (59.2 %)	0.019**	5.167
Female, n (%)	2 (11.8 %)	93 (40.8 %)		
Thai, n (%)	8 (47.1 %)	122 (53.5 %)	0.625**	0.772
Burmese, n (%)	9 (52.9 %)	106 (46.5 %)		

*Comparison of the two groups using the Mann–Whitney U test

**Comparison of the two groups using the Fisher’s exact test

Of the patients without a G6PD deficiency, 26 (11.4 %) were infected with *P. falciparum* and 202 cases (88.6 %) were infected with *P. vivax*. The percentage of *P. falciparum* infected and G6PD-deficient individuals and *P. falciparum* infected non-G6PD-deficient individuals was the same (~11 %). This clearly shows that having a G6PD deficiency does not protect against the *P. falciparum* infection. A significant association was observed between gender and G6PD status in patients with malaria (*P* < 0.05, *OR* = 5.167), with the incidence of G6PD deficiency being higher in males than in females. In this study, the absolute monocyte count was higher in patients with a G6PD deficiency (mean = 0.6 × 10^3^/μL) than those without a G6PD deficiency (mean = 0.33 × 10^3^/μL) (*P* < 0.05). However, no significant differences were observed between G6PD status and other hematological parameters, such as WBC count, neutrophil count, eosinophil count, basophil count, lymphocyte count, RBC count, Hb level, platelet counts, MCV, MCH, and MCHC (*P* > 0.05) (see Table 2). After adjusting for these parameters, a univariate analysis between G6PD status and hematological parameters showed that the absolute monocyte count was significantly different between those with and without a G6PD deficiency (*P* < 0.05) (see in Table 3). In addition, gender was significantly associated with G6PD status (*P* < 0.05), a multivariate analysis also showed that the difference in monocyte count between the two groups was still significant (*P* < 0.05). Among the patients with a G6PD deficiency, 11 cases (64.7 %) had a lower parasite density and six (35.3 %) had a moderate parasite density. Among the patients without a G6PD deficiency, 156 (68.45 %) had low parasitemia, 66 (28.95 %) had moderate parasitemia, and six (2.6 %) had high parasitemia.

**Table 2:** Hematological parameters of patients with malaria infection

Parameter	G6PD def.	Normal	*P*-value
	Mean ± SD	Mean ± SD	
Leukocyte (×10^3^/μL)	6.85 ± 1.37	6.21 ± 2.31	0.058
Neutrophil (×10^3^/μL)	4.77 ± 1.41	4.33 ± 1.98	0.929
Lymphocyte (×10^3^/μL)	1.2 ± 0.63	1.27 ± 0.9	0.313
Monocyte (×10^3^/μL)	0.6 ± 0.43	0.33 ± 0.28	0.013*
Eosinophil (×10^3^/μL)	0.2 ± 0.28	0.2 ± 0.75	0.552
Basophil (×10^3^/μL)	0.08 ± 0.06	0.07 ± 0.06	0.873
RBCs (×10^6^/μL)	4.5 ± 0.85	4.62 ± 0.72	0.845
Hb (g/dL)	12.16 ± 2.4	12.12 ± 1.96	0.862
Hematocrit (%)	36.76 ± 6.99	35.93 ± 5.55	0.668
MCV (fL)	82.02 ± 7.99	77.99 ± 8.7	0.093
MCH (pg/cell)	27.13 ± 2.9	26.36 ± 3.59	0.48
MCHC (g/dL)	33.08 ± 1.63	33.81 ± 1.31	0.069
RDW (%)	12.38 ± 1.86	12.7 ± 1.37	0.026*
Platelet (×10^3^/μL)	98.24 ± 71.81	83.82 ± 50.27	0.539

*P-value determined using the Mann–Whitney U test

**Table 3:** Univariate and multivariate analyses of G6PD status and hematological parameters adjusted by age, type of *Plasmodium* species, and parasite density of patients

Analysis type	Variable	G6PD def./normal	
		B*	*P*-value
Univariate	Monocyte	−2.152	0.001
	RDW	0.217	0.358
	Age	−0.004	0.78
	Gender	−1.642	0.032
	*P. falciparum*/*P. vivax*	−0.035	0.964
	Nationality	0.258	0.608
	Parasite density	0.167	0.751
Multivariate	Monocyte	−2.307	0.002
	RDW	0.361	0.150
	Age	−0.019	0.295
	Gender	−1.527	0.052
	Nationality	0.301	0.592
	*P. falciparum*/*P. vivax*	0.131	0.877
	Parasite density	0.412	0.491
	Constant	0.290	0.931

*unstandardized variables

## Discussion

A previous study showed that 20.8 % of the Thai and Burmese populations who reside in malaria-endemic areas (western, northern, northeastern, southern, eastern, and central regions) of Thailand are G6PD deficient [12]. This indicates that malaria and G6PD deficiency share similar geographical distributions. This study showed that 6.9 % of malaria-infected patients were diagnosed with a G6PD deficiency, which is lower than reported by previous studies conducted in southeast Iran (27 %) [13], Cambodia (13.9 %) [14], and southwest Ethiopia (7.3 %) [15]. However, it was higher than that reported in Eastern Indonesia (5.1 %) [16] and the Brazilian Amazon (4.5 %) [17]. A recent study conducted in the Thailand-Myanmar border area indicated that the overall frequency of G6PD deficiency determined using the fluorescence spot test was 13.7 %. Of those patients, almost 90 % had the Mahidol variant (487G > A) genotype, whereas the remaining subjects had the Chinese-4 (392G > T), Viangchan (871G > A), Açores (595A > G), Seattle (844G > C), or Mediterranean (563C > T) variants [18]. Another study focusing on the Thailand-Myanmar border area also found a high frequency of G6PD Mahidol (96.2 %; 38.1 % in the Thai population and 96.2 % in the Burmese population) [12]. The prevalence of G6PD deficiency in other areas of Thailand such as in Chiang Mai province was found to be lower (4 %) than along the Thailand-Myanmar border [19].

A significant association was observed between gender and G6PD status in patients with malaria (*P* < 0.05, *OR* = 5.167), with the incidence of G6PD deficiency being higher in males than in females. This is similar to the findings of other studies [14, 20], and is likely due to G6PD deficiency being an X-linked hereditary disease, with the male tending to be at greater risk than the female as the male has only one X-chromosome. Hemizygous males and homozygous normal females are most and least commonly affected, respectively. Heterozygous females have mixed G6PD normal and deficient RBCs, and their total G6PD enzyme activity and susceptibility to hemolysis depends on the balance between the expression of the normal and abnormal X-chromosomes [21]. A previous study reported that mutation in the *G6PD* gene gives boys substantial protection against severe malaria. The reason for this difference in protection is not clear, as it is not known how malaria parasites are affected by RBCs that lack G6PD activity [22]. Some epidemiological and clinical studies have shown that individuals who are heterozygous and/or hemi-/homozygous are at lower risk of developing severe malaria or succumbing to it [23]. These findings are defined as the ‘malaria/G6PD hypothesis’ [24], but the mechanism of protection remains under discussion.

The absolute monocyte count was higher in patients with a G6PD deficiency than those without a G6PD deficiency. The main cause of a high monocyte count is supported by previous studies that indicate that a certain percentage of circulating phagocytic cells containing hemozoin is strongly associated with parasitemia and severity of disease in malaria patients [25, 26]. Hemozoin-loaded monocytes are also functionally impaired as they do not produce oxidative burst and are unable to repeat the phagocytic process [27, 28]. In addition, G6PD-deficient red cells will lyse upon oxidative stress produced by the parasites, and thus send signals to be phagocytosed more than normal red cells. This will generally explain why malaria is only mild or moderate and not severe in patients with a G6PD deficiency.

Among the patients with a G6PD deficiency, 11 cases (64.7 %) had a lower parasite density and six (35.3 %) had a moderate parasite density. Among the patients without a G6PD deficiency, 156 (68.45 %) had low parasitemia, 66 (28.95 %) had moderate parasitemia, and six (2.6 %) had high parasitemia. This means that patients with a G6PD deficiency exhibit low and moderate parasitemia, whereas patients without a G6PD deficiency exhibit low, moderate, and high parasitemia. This is likely caused by the G6PD deficiency protecting against malaria, however, this study cannot conclude the role of G6PD deficiency against malaria because only a small number of patients with a G6PD deficiency was recruited and studied. Previous studies have also detected a lower parasite count in G6PD-deficient patients [10, 29, 30].

Awareness of high prevalence of G6PD deficiency was from potential hemolytic reactions due to treatment of malaria with primaquine. Primaquine is the only generally available antimalarial drug that prevents relapse in vivax and ovale malaria, and the only one that prevents potent gametocytocidal activity in falciparum malaria. Primaquine can cause dose-dependent AHA that is greater in more severely deficient G6PD variants; AHA can be potentially life threatening, but primaquine-related deaths are very rare [31, 32]. This toxicity is a significant public health concern because G6PD deficiency affects approximately 400 million people worldwide who live mostly in malaria-endemic countries, where the median G6PD deficiency allele prevalence was 8 % [33]. Aside from G6PD deficiency, patients with homozygous HbEE have a higher chance of *P. falciparum* infection compared to those with normal HbAA (odds ratio [*OR*] = 5.0) [34].

In addition to treatment of malaria with primaquine, drug resistance has remained the greatest challenge to malaria control. Despite a large number of antimalarial drugs available, there is no perfect drug; all have limitations such as poor compliance, side effects, toxicity, and/or resistance. The support from international organizations and governments of some endemic countries should be optimally exploited in various approaches to drug research and development in order to overcome the burden of malaria [35].

This study had several limitations: a small number of patients with a G6PD deficiency; could not obtain data about diseases affecting hematological parameters; and the fluorescent spot test is not a confirmatory test for G6PD. However, the results of this study still provide valuable information relating to G6PD deficiency in patients infected with malaria in endemic regions of Thailand.

## Conclusion

This study showed a prevalence of G6PD deficiency, which becomes apparent from potential hemolytic reactions due to treatment of malaria with primaquine. The study also supported the notion that patients with G6PD deficient-RBCs had no protection against *P. falciparum* infection. In addition, malaria patients with a G6PD deficiency had higher monocyte counts than those without a G6PD deficiency, and this was independent of age, gender, nationality, *Plasmodium* species, and parasite density. These findings will help to recognize and diagnose malaria patients with a G6PD deficiency, as well as to identify the risks and protective factors against malaria in endemic areas.

## Additional file

Additional file 1: Multilingual abstracts in the five official working languages of the United Nations. (PDF 418 kb)

## Abbreviations

AHA: acute hemolytic anemia
CBC: complete blood count
G6PD: glucose-6-phosphate dehydrogenase
Hb: hemoglobin
MCH: mean corpuscular hemoglobin
MCHC: mean corpuscular hemoglobin concentration
MCV: mean corpuscular volume
RBC: red blood cell
RDW: red cell distribution width
WBC: white blood cell
