# Supplementary material for: Prevalence and hematological indicators of G6PD deficiency in malaria-infected patients
Source: Infect Dis Poverty. 2016 Apr 25;5:36. doi: 10.1186/s40249-016-0130-0 (PMC4843198; doi:10.1186/s40249-016-0130-0)
Supplement: Additional file 1: — Multilingual abstracts in the five official working languages of the United Nations. (PDF 418 kb) [file 40249_2016_130_MOESM1_ESM.pdf]

Translation of the abstract into the five official working languages of the United Nations

انتشار عوز G6PD ومؤشراته الدموية لدى المرضى المصابين بعدوى الملاريا

ماناس كوتيبوي، كونتيدا يوثايسر، بوكدي فون فوينش، نوويل فويكلام

موجز

الخلفية: تهدف هذه الدراسة إلى تقييم مدى انتشار وتغيرات المعايير الدموية لدى مرضى الملاريا الذين يعانون من عوز نازعة خيدروجين الغلوكوز – 6- فسفات (G-6-PD)، في المنطقة الغربية من تايلند، وهي منطقة موبوءة بالملاريا.

الطرائق: جُمعت البيانات التي تتعلق بالمرضى الذين دخلوا إلى المستشفى بين عامي 2013 و2015. استخرجت الخصائص السريرية والاجتماعية الديموغرافية مثل العمر والجنس، والتشخيص وقت القبول، ونتائج الفحوص الطفيلية من السجلات الطبية لوحدة المختبر في مستشفى Phop Phra في مقاطعة تاك، تايلاند. جُمِعَت عينات من الدم الوريدي وقت الدخول إلى المستشفى لتقرير عوز G6PD بواسطة اختبار البقعة الومضاني ولتحري وجود طفيليات الملاريا باحتبار الفيلم الثخين والفيلم الرقيق. كما جمعت بيانات أخرى مثل تعداد الدم الكامل وكثافة الطفيلي وخضعت للتحليل.

النتائج: من بين 245 حالة ملاريا، شخّصت 28 حالة (11.4%) على أنها عدوى بالمتصورة المنجلية *Plasmodium falciparum*، و217 حالة (88.6%) على أنها عدوى بالمتصورة النشيطة *P. vivax*. كان 17 مريضا (6.9%) يعانون من عوز G6PD و228 مريضا (93.1%) لا يعانون من عوز G6PD. كان معدل انتشار عوز G6PD بين الذكور أعلى منه بين الإناث ($P<0.05$، نسبة الخطأ = 5.167). من بين المرضى الذين يعانون من عوز G6PD، كان اثنان (2) منهما (11.8%) مصابين بعدوى المتورة المنجلية، في حين كان الباقون مصابين بعدوى المتصورة النشيطة. لدى مرضى الملاريا المصابين بعوز G6PD عدد أكبر من الكريات البيضاء الوحيدة (0.6×10^3/ميكرولتر) مقارنة بغير المصابين بعوز G6PD (0.33×10^3/ميكرولتر) ($P<0.05$، نسبة الخطأ = 5.167). كما أكد التحليل أحادي المتغير والتحليل متعدد المتغيرات أن مرضى الملاريا الذين يعانون من عوز G6PD لديهم عدد أكبر من الوحيدات. وقد كان الارتباط بين حالة G6PD وتعداد الوحيدات مستقلا عن العمر، الجنس، الجنسية، نوع المتصوّرة، وكثافة الطفيلي ($P<0.005$).

الاستنتاج: أظهرت هذه الدراسة انتشارا لعوز G6PD في منطقة موبوءة بالملاريا. كما دعمت هذه الدراسة أيضا التأكيد على أن المرضى الذين لديهم خلايا دم حمراء معوزة الـ G6PD لم تتوفر لديهم حماية ضد عدوى المتصورة المنجلية. بالإضافة إلى ذلك، كان عدد الوحيدات أكبر لدى مرضى الملاريا المصابين بعوز G6PD مقارنة بغير المصابين بعوز G6PD. ستساعد هذه النتائج على معرفة وتشخيص مرضى الملاريا الذين يعانون من عوز G6PD، إضافة إلى تحديد المخاطر والعوامل الواقية ضد الملاريا في المناطق الموبوءة.

Translated from English version into Arabic by Lina SM, through

TRANSLATORS
WITHOUT BORDERS

疟疾患者中葡萄糖-6 磷酸脱氢酶（G6PD）缺乏症的流行情况与血液学指标

Manas Kotepui, Kwuntida Uthaisar, Bhukdee PhunPhuech, Nuoil Phiwklam

摘要

引言：本研究目的在于评估泰国西部疟疾流行区的疟疾患者中 G6PD 缺乏症的流行情况和血液学参数的变化。

方法：收集了泰国 Tak 省 Phop Phra 医院 2013-2015 年疟疾患者资料，包括从该院实验室的医疗记录中取得的年龄、性别、入院诊断和寄生虫学诊断结果等临床和社会人口统计学数据。患者入院时采集了静脉血样，采用荧光点测试检测 G6PD 缺乏，使用厚薄血膜镜检疟原虫。同时收集和分析其他数据，如全血细胞计数和原虫密度。

结果：在 245 例疟疾病例中，28 例(11.4%)确诊为恶性疟，217 例(88.6%)为间日疟。17(6.9%)例患有 G6PD 缺乏症。男性疟疾患者的 G6PD 缺乏症患病率比女性高(*P*<0.05，*OR*=5.167)。在罹患 G6PD 缺乏症的疟疾患者中，2 例(11.8%)为恶性疟，其他均为间日疟。罹患 G6PD 缺乏症的疟疾患者的单核白血球数(0.6×10^3/μL)比未患该病的疟疾患者高(0.33×10^3/μL) (*P*＜0.05, *OR*=5.167)。单变量分析和多变量分析均证实罹患 G6PD 缺乏症的疟疾患者的单核白血球数更高。G6PD 状态和单核白血球数之间的关联独立于年龄、性别、国籍、疟原虫种类以及寄生虫密度(*P*＜0.005)。

结论：本研究揭示了疟疾流行地区的 G6PD 缺乏症的患病情况。本研究同样支持以下结论，即拥有 G6PD 缺乏红细胞的患者对恶性疟原虫感染没有保护作用。此外，与未患 G6PD 缺乏症的疟疾患者相比，患有该病的疟疾患者单核白血球数更高。这些发现将有助于识别和诊断患有 G6PD 缺乏症的疟疾患者，以及在流行区识别疟疾危险因素和保护因素。

Translated from English version into Chinese by Yan-Qi Zhu, edited by Pin Yang, through

CHINESE CENTER FOR DISEASE CONTROL AND PREVENTION
NATIONAL INSTITUTE OF PARASITIC DISEASES

Prévalence et indicateurs hématologiques du déficit en G6PD chez les patients infectés par le paludisme

Manas Kotepui, Kwuntida Uthaisar, Bhukdee PhunPhuech, Nuoil Phiwklam

Résumé

Contexte : Cette étude visait à évaluer la prévalence et l’altération des paramètres hématologiques chez les patients atteints du paludisme présentant un déficit en glucose-6-phosphate déshydrogénase (G6PD) dans une région de paludisme endémique de l’ouest de la Thaïlande.

Méthodes : Nous avons recueilli les données de patients atteints du paludisme hospitalisés entre 2013 et 2015 en recherchant leurs caractéristiques cliniques et sociodémographiques (âge et sexe, diagnostic à l’admission, résultats de parasitologie) dans les dossiers médicaux du laboratoire de l’hôpital Phop Phra, dans la province de Tak en Thaïlande. Des échantillons de sang veineux ont été recueillis au moment de l’hospitalisation afin de déterminer une carence en G6PD par le spot-test de Beutler et de déceler le parasite agent du paludisme par examen au microscope sur frottis et goutte épaisse. D’autres données, comme la NFS et la densité de parasites, ont également été recueillies et analysées.

Résultats : Parmi les 245 cas de paludisme, 28 (11,4 %) ont été diagnostiqués comme des infections à *Plasmodium falciparum* et 217 (88,6 %) comme des infections à *P. vivax*. Dix-sept patients (6,9 %) présentaient un déficit en G6PD et 228 (93,1 %) n’avaient pas ce déficit. Les hommes déficients en G6PD étaient plus nombreux que les femmes (*P*＜0,05, *OR*=5,167). Deux des patients déficients en G6PD (11,8 %) étaient infectés par *P. falciparum* et tous les autres par *P. vivax*. Les patients atteints du paludismes et déficients en G6PD avaient une numération monocytaire plus élevée (0,6×10^3/µl) que ceux qui ne présentaient pas ce déficit (0,33×10^3/µl) (*P*＜0,05, *OR*=5,167). Des analyses uni- et multivariées ont également confirmé que les patients atteints du paludisme présentant un déficit en G6PD avaient une numération monocytaire élevée. L’association entre le statut de G6PD et la numération monocytaire était indépendante de l’âge, du sexe, de la nationalité, de l’espèce de *Plasmodium* et de la densité parasitaire (*P*＜0,005).

Conclusion : L’étude a mis en évidence une prévalence du déficit en G6PD dans une zone de paludisme endémique. Elle a également apporté des arguments à l’appui de l’affirmation selon laquelle les patients dont les

érythrocytes sont déficients en G6PD ne sont pas protégés contre l'infection par *P. falciparum*. En outre, les patients atteints du paludisme et déficients en G6PD avaient une numération monocytaire plus élevée que ceux qui ne présentaient pas ce déficit. Ces résultats aideront à reconnaître et diagnostiquer les patients atteints du paludisme et déficients en G6PD et contribueront à identifier les facteurs de risque et de protection contre le paludisme dans les régions d'endémie.

Translated from English version into French by Suzanne Assenat, through

Распространённость и гематологические показатели недостаточности глюкозо-6-фосфатдегидрогеназы у пациентов, инфицированных малярией.

Манас Котепуи, Квунтида Утхайсар, Бхукдее Пхун Пхуех, Нуоил Пхивклам

Общая информация

Ситуация: Это исследование направлено на то, чтобы оценить распространённость и изменение гематологических параметров у больных малярией с недостаточностью глюкозо-6-фосфатдегидрогеназы в западной части Таиланда, являющейся эпидемическим очагом малярии.

Методы: Были собраны данные о пациентах, госпитализированных с малярией в период с 2013 по 2015 год. Клинические и социодемократические показатели, такие как возраст и пол, диагноз при поступлении и результаты паразитологических исследований были получены из медицинских записей лаборатории больницы ФорФра в провинции Так, Таиланд. В момент поступления в больницу были взяты образцы венозной крови, чтобы установить недостаточность глюкозо-6-фосфатдегидрогеназы при помощи флуоресцентного спот-теста и выявить малярийных паразитов при исследовании густой капли и мазка крови. Были также собраны и проанализированы другие данные, такие как развёрнутый анализ крови и плотность паразитов.

Результаты: Из 245 случаев заболевания малярией 28 (11,4%) были диагностицированы как инфекции *Plasmodium falciparum* и 217 случаев (88,6%) - как инфекции *P.vivax*. У семнадцати (6,9%) пациентов наблюдалась недостаточность глюкозо-6-фосфатдегидрогеназы, а у 228 (93,1%) пациентов не было недостаточности глюкозо-6-фосфатдегидрогеназы. Недостаточность глюкозо-6-фосфатдегидрогеназы встречалась чаще у пациентов мужского пола, чем у пациентов женского пола ($P<0.05$, OR=5.167). Двое (11,8.%) из пациентов, страдающих недостаточностью глюкозо-6-фосфатдегидрогеназы, были инфицированы *P. falciparum,* в то время как остальные были инфицированы *P. vivax.* У пациентов с недостаточностью глюкозо-6-фосфатдегидрогеназы было более высокое число моноцитов (0.6×10^3/μL), чем у пациентов, не имеющих недостаточности глюкозо-6-фосфатдегидрогеназы (0.33×10^3/μL) ($P<0.05$, OR=5.167). Однофакторный и многофакторный анализы также подтвердили, что больные малярией, страдающие недостаточностью глюкозо-6-фосфатдегидрогеназы, имеют высокое число моноцитов. Связь между недостаточностью глюкозо-6-фосфатдегидрогеназы и числом моноцитов не зависела от возраста, пола, национальности, вида *Plasmodium* и плотности паразитов ($P<0.005$).

Вывод: Это исследование показало распространённость недостаточности глюкозо-6-фосфатдегидрогеназы

в области, эндемичной по малярии. Это исследование также подтвердило утверждение, что пациенты с эритроцитами с недостаточностью глюкозо-6-фосфатдегидрогеназы не защищены от инфекции *P. falciparum.* Кроме того, больные малярией, страдающие недостаточностью глюкозо-6-фосфатдегидрогеназы, имеют более высокое число моноцитов, чем те, у кого нет недостаточности глюкозо-6-фосфатдегидрогеназы. Эти данные помогут опознать и диагностицировать больных малярией с недостаточностью глюкозо-6-фосфатдегидрогеназы, а также выявить риски и факторы защиты от малярии в эндемических областях.

Translated from English version into Russian by Tatiana Glazina, through

Prevalencia e indicadores hematológicos de la deficiencia de G6PD en pacientes con malaria

Manas Kotepui, Kwuntida Uthaisar, Bhukdee PhunPhuech, Nuoil Phiwklam

Resumen

Antecedentes: El objetivo del presente estudio fue evaluar la prevalencia y alteración de los parámetros hematológicos en pacientes con malaria con una deficiencia de glucosa-6-fostato deshidrogenasa (G6PD) en la región occidental de Tailandia, una región endémica para la malaria.

Métodos: Se recolectó información de pacientes con malaria hospitalizados entre los años 2013 y 2015. Se extrajeron características clínicas y sociodemográficas, como edad y sexo, diagnóstico al ingreso, y resultados parasitológicos de las historias clínicas de la unidad de laboratorio del Hospital Phop Phra en la provincia de Tak, en Tailandia. Se recolectaron muestras de sangre venosa al momento del ingreso de los pacientes al hospital para determinar la deficiencia de G6PD por prueba de mancha fluorescente y detectar parásitos de malaria por examen de película fina y gruesa. También se recolectó y analizó otro tipo de información, como recuento sanguíneo completo y densidad parasitaria.

Resultados: De los 245 casos de malaria, 28 (11,4%) fueron diagnosticados como infecciones por *Plasmodium falciparum* y 217 casos (88,6%) fueron diagnosticados como infecciones por *P. vivax*. Diecisiete (6,9%) pacientes tenían una deficiencia de G6PD y 228 (93,1%) pacientes no tenían deficiencia de G6PD. La prevalencia de pacientes masculinos con deficiencia de G6PD fue más elevada que aquella de pacientes femeninos ($P<0,05$, $OR=5,167$). Entre los pacientes con deficiencia de G6PD, dos (11,8%) tenían infección por *P. falciparum,* mientras que el resto tenía infección por *P. vivax*. Los pacientes con malaria con deficiencia de G6PD tienen recuentos de monocitos más elevados ($0,6\times10^3/\mu L$) que aquellos sin deficiencia de G6PD ($0,33\times10^3/\mu L$) ($P<0,05$, $OR=5,167$). Los análisis univariante y multivariante también confirmaron que los pacientes con malaria con deficiencia de G6PD tenían recuentos más elevados de monocitos. La correlación entre el estado de la G6PD y el recuento de monocitos era independiente de la edad, sexo, nacionalidad, especie de *Plasmodium* y densidad parasitaria ($P<0.005$).

Conclusión: Este estudio mostró una prevalencia de deficiencia de G6PD en una zona endémica para la malaria. Este estudio también sustentó la afirmación que los pacientes con glóbulos rojos con deficiencia de G6PD no están protegidos contra la infección por *P. falciparum*. Asimismo, los pacientes con malaria con deficiencia de G6PD

tienen un recuento de monocitos más elevado que aquellos sin deficiencia de G6PD. Estos hallazgos ayudarán a reconocer y diagnosticar pacientes con malaria con deficiencia de G6PD y a identificar los riesgos y factores de protección contra la malaria en regiones endémicas.

Translated from English version into Spanish by Maria Alejandra Aguada, through

TRANSLATORS WITHOUT BORDERS
